# Evaluating nursing student confidence after sexual health training workshop

**DOI:** 10.1186/s12912-026-04509-y

**Published:** 2026-03-05

**Authors:** McKeaveney Clare, McKenna Niall, John Nimmy, Galeotti Martina

**Affiliations:** School of Nursing and Midwifery, Queens University Belfast, Belfast, UK

**Keywords:** Sexual and reproductive health, WHO, Trauma informed care, Educational intervention, 6Ps framework, Mixed- methods approach

## Abstract

**Objectives:**

This study adopted a mixed-methods approach to evaluate the impact of a sexual health training session on undergraduate nursing students’ confidence, knowledge, and comfort in discussing and assessing patients’ sexual and reproductive health. The intervention was framed using the 6Ps model and grounded in trauma-informed care principles.

**Methods:**

The study was conducted at Queen’s University Belfast, involving final-year adult nursing students. A total of 98 third-year undergraduate adult nursing students participated in the pre- and post-intervention survey. Attendance at the training session was part of the curriculum, while survey participation was voluntary.

**Results:**

Most students (*n* = 81) had not received prior training in sexual health and reported limited opportunities to discuss sexual health during clinical placements. Baseline data revealed low confidence and knowledge in this area, with identified barriers including embarrassment, lack of privacy, and cultural/religious concerns. Following the training, statistically significant improvements were observed across all measured domains: comfort (Z= -7.431 to -8.118; *p*<.001), confidence (Z= -7.434 to -8.278; *p*<.001), and knowledge (Z= -7.606 to -7.996; *p*<.001). Open-text responses indicated enhanced awareness of holistic assessment, greater familiarity with sensitive topics such as Chemex and HIV transmission, and improved skills in initiating conversations with patients.

**Conclusion:**

A short educational intervention significantly enhanced nursing students’ knowledge and confidence in sexual and reproductive health assessment. Findings underscore the need for further curriculum development and integration of trauma-informed, evidence-based sexual health training using the 6Ps framework in undergraduate nursing programmes.

**Clinical trial number:**

Not applicable.

**Supplementary Information:**

The online version contains supplementary material available at 10.1186/s12912-026-04509-y.

## Introduction

 According to the World Health Organisation (WHO) [1] sexual health is defined as a state of physical, emotional, mental, and social well-being regarding sexuality; it is not simply the absence of disease or dysfunction. WHO emphasises that for sexual health to be attained and maintained, all individuals’ sexual rights must be acknowledged, protected and fulfilled. Sexuality is considered a fundamental aspect of holistic care [2]. It forms part of the Activities of Daily Living model [3] which provides a framework for nursing care and assessment. It is important for personal physical, mental, psychological, emotional, and ethical well-being as well as interpersonal relationships [4]. Therefore, addressing patients’ sexuality is important, necessitating a multidisciplinary approach and is not the responsibility of a single professional [5].

The 6P model incorporates any history of Sexually Transmitted Infections (STIs), protection, pregnancy and fertility and P plus- pleasure, problems and Pride-LGBTQ into sexual health assessment [6]. Experts in undergraduate education recognise that principles of sexual health assessment and the 6 Ps should be a mandatory component of sexual health education for HCPs [7, 8]. It is well documented that healthcare professionals (HCP) do not routinely assess patients’ sexual health [9]. Barriers such as embarrassment, lack of knowledge and the perceived stigma of discussing sexual health needs reduce HCP confidence when discussing these with patients [2].

Similarly, others reported student nurses feel uncomfortable addressing sexual health and are reluctant to initiate a conversation [10]. This emphasises the need for further intervention to enhance confidence and improve sexual health assessment skills in the nursing curricula supporting the importance of this study. Several studies have developed interventions focused on the Sexual Health of LGBTQ populations and/or women’s health issues, with only a few interventions incorporating skills based on the comprehensive 6P framework [6]. Another study evaluated a mandatory sexual health course with first-year undergraduate medical students through pre and post Objective Structured Clinical Examinations showing statistically significant improvements in communication skills in sexuality [11]. Further, it was found that a mandatory sexual-health course can improve graduate nurses’ knowledge, preparedness, comfort, and confidence in delivering informed sexual healthcare was also effective [12]. Many curricula only focus on increasing knowledge on sexual health rather than equipping nurses to feeling comfortable in initiating this conversation with patients [12, 13].

To the best of our knowledge, there are limited interventions regarding sexual health education in undergraduate nursing education. A few curricula have experimentally tested sexual healthcare education on nursing students’ knowledge, attitudes, and improvement in addressing patient’s sexual healthcare needs [4, 14]. This highlights a gap in the current evidence available. Ultimately emphasising the need to evaluate and implement sexual health education sessions among nursing students using the 6P model with an overall goal to evaluate and establish a connection between confidence and knowledge in sexual health.

The general aim of this study was to evaluate the effectiveness of a sexual health session among undergraduate nursing students on levels of confidence, knowledge and awareness to assess and discuss sexual health needs in practice. In this light the following objectives were addressed:

Educational objectives:

- To disseminate evidence-based research knowledge to students;
- To increase students’ knowledge and confidence regarding sexual health in practice;

Research objectives:

- To increase students’ level of comfort talking about sexual health issues patients;
- To increase students’ confidence in talking about sexual health with patients;
- To identify educational gaps in sexual health learning in nursing professional curriculum.

## Methods

The study employed a primarily quantitative survey, complemented by optional open-text questions to capture qualitative insights. This represents an embedded mixed-methods approach, where the qualitative data provide context and elaboration for the quantitative findings. This project assessed pre- and post- intervention survey which also contained open ended questions. Quantitative and qualitative data was collected simultaneously but analysed separately.

### Study setting

The study is based in the United Kingdom (UK) where nursing programmes are validated by the Nursing and Midwifery Council (NMC). At Queen’s University Belfast (QUB), nursing students undertake nine clinical placements over the course of their three-year programme. These placements provide exposure to a wide variety of clinical environments, supporting the development of well-rounded clinical competence in line with NMC standards [15].

Student nurses may encounter opportunities to discuss sexual health with patients in a variety of settings, including, but not limited to, the emergency department, genitourinary medicine (GUM) clinics, community health services, and medical wards. Given the WHO’s broad definition of sexual health, there are numerous clinical contexts in which conversations related to sexual health are both appropriate and relevant. We acknowledge that sexual health may not be routinely discussed in certain environments; however, at the training stage it is essential to ensure that nurses develop a broad and comprehensive understanding of the importance of addressing sexual health as part of a holistic assessment.

This study was conducted within a core module entitled “Specialist and Complex Care” which explores different chronic conditions and their nursing management. This includes topics such as chronic kidney diseases, endometriosis, and cancer. The intervention was developed as part of the teaching material of the module.

## Design

### Informed consent and participants’ wellbeing

Participants were provided with an information sheet and consent form before starting the survey and could not continue to complete the survey without providing consent. Due to the sensitive nature of the topic, participants were encouraged to leave the lecture/workshop if feeling distressed. Further, all students were encouraged not to take part in the research study if they did not wish to. Their emotional distress was a priority, and therefore, all participants were provided with a debrief sheet with relevant support contact information whether they took part in the study or not.

### Ethical approval

This study was approved by the Faculty of Medicine, Health and Life Sciences Research Committee at QUB (MHLS 24_116). All participants completed a consent form before taking part in the study. This research study was conducted in accordance with the Declaration of Helsinki.

### Rigour

Lincoln and Guba framework was applied to ensure rigour [16, 17]. The credibility and transferability of the study were enhanced by providing a thick description of the study settings, inclusion and exclusion criteria, and the processes of data collection and analysis [18]. MG and CM have extensive experience in conducting mixed-methods research. All lecturers involved in delivering the tutorial were fully briefed, and each received preparatory materials to ensure consistency in the workshop’s delivery. To maintain alignment across sessions, all tutors were also provided with an answer sheet, ensuring that the content presented was accurate and uniform. Dependability is commonly ensured through external audits [19]. The protocol of this research study has been peer-reviewed by two members of staff from QUB before submitting to the ethics panel. Reflexivity was applied throughout the research process by critically examining the research team experiences might influence the study. Multiple team meetings were held to discuss the study and ensure that the intervention was consistent.

### Intervention content

A 2-hour face to face lecture followed by a 2- hour face to face workshop was delivered among 3rd -year adult nursing students. The lecture was delivered 3 days prior the workshop, this is the standard practice for the module where student attend a lecture followed by a workshop. As part of their curricula all students attended the lecture and workshop. However, the completion of the pre-post questionnaire was not mandatory.

#### Lecture component

The lecture utilised the 6Ps Framework to enhance students’ awareness of sexual and reproductive health assessment within nursing practice. This framework supports a comprehensive evaluation of different domains while adopting a trauma-informed approach; trauma refers to experiences that cause intense physical and psychological stress reactions. It results from events or circumstances that are experienced by an individual as physically and/or emotionally harmful or threatening. The Clinician Guide for Trauma- Informed Care was used to inform the lecture [20].

At the start of the lecture, a definition of sexual health from the WHO was provided [21].Students were reminded that sexual health is a fundamental component of patient care. Due to time constraints and the module learning outcomes, the lecture focused specifically on sexual health and did not cover wider aspects of reproductive health. The 6Ps framework was explained to consider its domains in the holistic assessment (Partners, Practice, Past history of STIs, Protection, Pregnancy and Plus (Pleasure, Problems and Pride). However, Pregnancy and some of the Plus categories (Pleasure and Problems) were not discussed during this lecture but acknowledged as an important component of the assessment. For example, LGBTQ+ communities and other minority groups were acknowledged, emphasising the need for a non-judgemental, inclusive approach. Practitioners have a duty to recognise diversity [15] and ensure equal access to care (WHO). Populations at increased risk, such as GBMSM (gay, bisexual, and other men who have sex with men) and individuals from minoritised ethnic backgrounds, were discussed not to stigmatise but to highlight the importance of prioritising them with appropriate resources and targeted interventions.

The aetiology, pathophysiology, and treatment of HIV and the main sexually transmitted infections (STIs) were presented. Additionally, the session addressed common barriers that nurses may encounter when conducting sexual and reproductive health assessments, including embarrassment, lack of knowledge, and limited confidence.

The Roadmap to Trauma-Informed Care was discussed to highlight the importance of communicating sensitively with patients, recognising that conversations about sexual health may risk triggering or retraumatising individuals.

#### Workshop component

Following the lecture, students participated in a workshop (17–25 students per group) in which they discussed the holistic assessment of a patient presenting to a GUM clinic with symptoms suggestive of HIV. In preparation for the workshop, students were asked to answer a series of questions related to the case study. These prompts were designed to guide them through the 6Ps framework and support the use of trauma-informed care in their assessment.

The case study, summarised in Table 1, formed the basis of an interactive discussion facilitated by the lecturer. Students explored assessment considerations, communication strategies, and ways to remain sensitive and mindful when addressing potentially distressing topics. The workshop aimed to consolidate the lecture content, reinforce the importance of trauma-informed practice, and provide students with the opportunity to apply theoretical knowledge to a realistic clinical scenario. For more information about the content of the workshop and tutorial please refer to Table 1.

**Table 1:** Content of lecture and workshop

Lecture		
Themes	Content	6Ps domain
1. Sexual Health in Nursing	The WHO (2006) definition of sexual health was provided to students stressing the importance of including sexual health assessments within nursing. This was used to set the scene and adhere to a global definition of sexual health. This definition did not include reproductive health. The 6Ps framework was explained to consider its domains in the holistic assessment (Partners, Practice, Past history of STIs, Protection, Pregnancy and Plus (Pleasure, Problems and Pride). However, Pregnancy and some of the Plus categories (Pleasure and Problems) were not discussed during this lecture but acknowledged as an important component of the assessment. The prevalence of STIs and HIV both in the United Kingdom and Northern Ireland were outlined.	Partners & Practice
2. Impact of Sexual Health on Patients	The overview of the impact of sexual health on patients was discussed including: how minorities such as GBMSM and African communities are at higher risk of STIs; the impact on sexual health assessments on patients’ mental health was explored; examples of stigma and embarrassment in engaging with sexual health services.	Past history of STis, practices, partners
3. Effective Communication and Sexual Health	The Trauma-Informed Care Roadmap was used to explain how trauma-informed care can be applied to the sexual health assessment. Students were taught the importance of being mindful of the risks of re-traumatising or triggering patients. Practical examples were provided to demonstrate how procedures should be explained and how to recognise signs of distress. Furthermore, communication barriers were explored, including issues related to privacy, religious background, and stigma when initiating conversations with patients.	Past history of STIs, practices, partners
4. Common STIs and HIV	Pathophysiology of HIV and common STIs were explained. Specifically, prevention, diagnosis and treatment of HIV, Chlamydia, Syphilis, Gonorrhoea were discussed.	Past history of STIs, Protection
6. Supporting patients	The importance of providing educational information to patients and signpost to additional services such as genitourinary clinics was stressed. The lecture contained educational material shared in the GUM clinic website (https://sexualhealthni.info/stis/)	Partners, Pride & Problems, Past history of STis, practices, partners
Workshop		
**Case scenario**	**Student activity**	**6Ps domains**
You have allocated a placement in the local Genito-Urinary Medicine (GUM) Clinic. As part of your development plan, your Practice Assessor has asked you to develop your skills in assessing the clients that attend the clinic.	1. Critically examine the components of a holistic assessment for the range of clients that may present to the GUM clinic.	Students had to use the 6Ps framework and The Trauma Informed Road Map to answer the questions in class. The lectures used an answer sheet to ensure all the questions were answered and students were provided the same information.
Zakir (Zak) Noorani is a 27-year-old man who has booked into the GUM clinic for an appointment. During your sexual health assessment, you confirm the following. Zac is presenting dysuria, and urethral discharge and states that he hasn’t attended a sexual health check in at least 12 months. His general health has been good although he reports having a ‘bad flu’ 8 weeks ago. Zakir states that he is bi-sexual and has engaged in casual sex on a regular basis since he attended University. He confirms that in the recent past that he has participated in chemsex parties and would consume alcohol on a regular basis. In the past (last 3 months) he confirms that, he has had a short-term partner (male) but this relationship has recently ended. While initially on PrEP Zak hasn’t taken his medication in several weeks. Coming from a large family circle, Zak has limited contact with his parents/siblings as they hold strong religious beliefs and disapprove of his sexuality. Zak works as a teacher in a local secondary school	2. Critically discuss the range of STI’s that Zak is at risk of given his sexual history – detail the potential clinical manifestations of these infections. What factors predispose Zac in having increased risk of infection? 3. Examine the barriers that may have delayed Zac in attending sexual health services or adopting ‘safe sex’ practices. 4. Given Zak’s presentation appraise the routes of HIV transmission and outline the pathophysiology of HIV and its impact on the immune system. What impact may a diagnosis of HIV have on an individual following HIV diagnosis. 5. Analyse the health promotion/education advice that ought to be provided to Zac following his diagnosis to minimise the risk of complications of HIV. Detail key treatment approaches in the management of his HIV positive status.	

### Inclusion criteria

Third year undergraduate nursing students, who were enrolled on a nursing degree at QUB were included.

### Recruitment

Two cohorts of students of students (approximately 300 students) were contacted via email by the module coordinators to inform them about the study. Participants information sheet was uploaded among the module resource prior to the lecture and workshop.

### Data collection

Data was collected from all participants who opt-in to complete a pre- and post-test questionnaire online. The survey included multiple questions adapted from previous studies [22, 23] and created by the research team-.

Data collection was conducted with two student cohorts (a September and a February intake). Data collection took place when students were timetabled to attend class as part of their compulsory module. The first data collection period occurred in December 2024, and the second in May 2025. At the first time point, the lecture was delivered, and the questionnaire was made available for one day. The workshop took place two days later, and data were collected immediately after its completion. The post-workshop survey remained open for 24 h.

The questionnaire included demographic questions such as age, gender, and previous training on sexual and reproductive health. Specifically, it evaluated how comfortable students were to discuss sexual health with patients; how confident they would be to talk to patients about sexual health; and their level of knowledge such as sexually transmitted infections. Five open-ended questions were included to explore students’ perception of the importance of discussing sexual health issues with patients and the main barriers in doing so. Additionally, students were asked to indicate the impact that the educational material delivered had on their knowledge and confidence around the topic. Finally, students were asked to discuss the areas, if any, were they lack in confidence and knowledge around sexual health (see appendix 1 for more information about the survey questions).

Students were invited to scan a QR code both after the lecture and after the workshop in order to complete the questionnaires. Data collection was undertaken by a member of the team who was not involved in analysing the data, ensuring separation between data collection and analysis. Data collection was made fully anonymous once pre and post test scores were merged. Student ID numbers were used solely for the purpose of pairing the pre- and post-questionnaires. Once the pairing was completed, the ID numbers were immediately discarded to maintain anonymity.

Additionally, the analysis of the research data was performed by CM, who is not involved in the marking of students’ exams. This ensured that participation, or non-participation, had no influence on academic outcomes.

Data analysis was conducted by a team member who does not teach the students directly, further supporting impartiality and reducing the risk of bias.

### Data analysis

#### Quantitative

Pre and post-test analyses were conducted using Wilcoxon signed ranked (paired) test to assess the differences before and after the intervention. Students’ responses on a 5-point Likert scale (1 = Very unlikely to 5 = very likely) were treated as ordinal variables. Wilcoxon signed-rank tests were deemed appropriate for pre- and post-intervention scores. A p-value of less than 0.05 was considered statistically significant. Effect sizes (r) were calculated for all analyses to provide an indication of the magnitude of observed differences, in addition to reporting p-values.

#### Qualitative data

Open text responses were downloaded from Pavlovia, uploaded on Excel and content analysis was performed, highlighting codes and themes using different colours. The occurrences of similar words or sentences were grouped [24]. The survey structure was used to code students’ responses under three main themes: barriers, knowledge and confidence. No quotes were added to confidence as students did not discuss narratively how the intervention had impacted their level of confidence. Only manifest data analysis was conducted, and themes were not interpreted [25]. Qualitative coding was conducted by MG and discussed between CM and MG to enhanced rigour.

#### Integration of quantitative and qualitative findings

This study was reported in line with the GRAMMS guidance to ensure transparent presentation of mixed-methods design, integration, and interpretation [26]. An explanatory mixed-methods approach was employed, as this design was deemed most appropriate for elucidating the relationships between the quantitative trends and the qualitative insights, and for providing a comprehensive interpretation of the data [27]. Integration occurred at the interpretation stage, where qualitative themes were used to explain and extend quantitative trends regarding choice, communication, and emotional outcomes. The thematic framework provided a systematic means of presenting and interpreting the findings. Qualitative data were integrated within the analysis to complement and expand upon the quantitative results, thereby offering a more in-depth understanding of participants’ experiences [28].

## Results

### Demographic characteristics

A total of 98 out of 300 3rd -year adult nursing students participated in the pre and post survey, representing approximately one-third of the sample. Research suggests reliable estimates can still be obtained with response rates in this range, particularly for exploratory or educational research purposes [29]. Table 2 shows most were aged 18–24 years (*n* = 69), followed by those aged 25–30 (*n* = 9), 31–36 (*n* = 10), 37–40 (*n* = 4) and 41–50 (*n* = 6). Most students were female (*n* = 94).

**Table 2:** Sociodemographic information

Characteristic	Category	*N*	%
Age	18–24	69	70.4
	25–30	9	9.2
	31–36	10	10.2
	37–40	4	4.1
	41–50	6	6.1
Sex	Female	94	95.8
	Male	4	4.2
Nursing Field	Adult	98	100%

### Findings

Data was organised in three key findings. These were (1) Barriers (2) Knowledge and (3) Confidence. The findings present the quantitative data and have been enhanced by quotes.

### Finding 1. Barriers

**Fig. 1:**
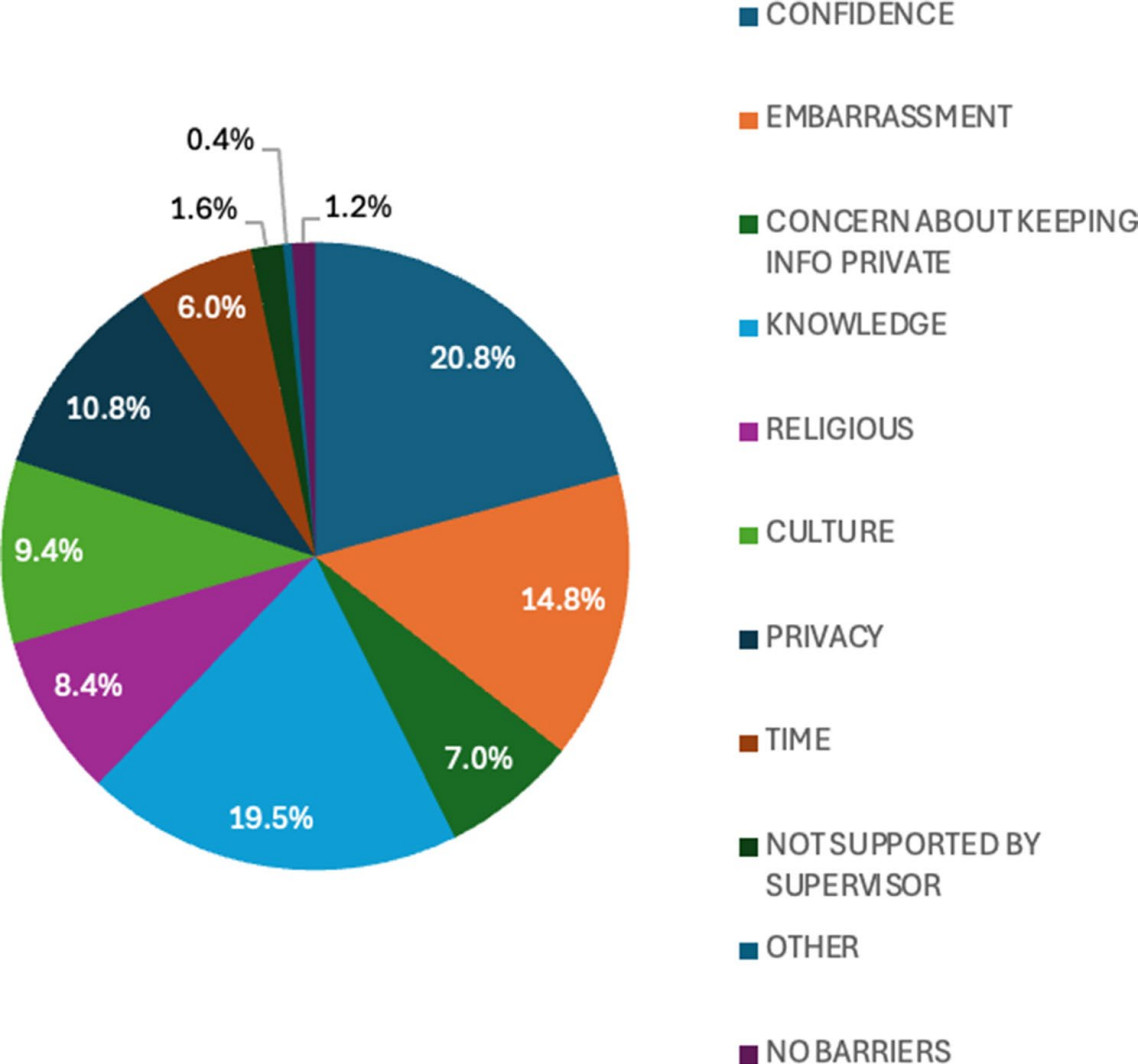
Barriers to initiating a conversation on sexual health with patients

Figure 1 shows that student nurses reported on several barriers to initiating conversations about sexual health with patients. Confidence and lack of knowledge (20.8%, 19.5% respectively) were the most frequently listed, followed by embarrassment (14.8%), privacy (10.8%), culture (9.4%), religious reasons (8.4%), concern about confidentiality (7.0%), time (6.0%) and feeling unsupported by a supervisor (1.6%), while some reported having no barriers (1.2%). Other barriers reported included being much younger than the patient and it not being relevant to their role (0.4%).

Further details on the lack of training were also identified in this study. Most 3rd -year nursing students had not received any training on sexual health to date (*n* = 81), whereas 17 reported they did receive some training. Training opportunities were referred to during their previous schooling (e.g. ‘In secondary school, we had one class based on sex’), university education (e.g. ‘public health group project’) and within previous jobs (e.g. ‘In my job whenever I worked in a sexual health clinic). Most students reported they did not have an opportunity to discuss sexual health with patients while on clinical placement (n = 76).

The remaining twenty-two students reported they did discuss sexual health with patients while on clinical placement and identified several clinical areas including most often, Genitourinary ‘GUM’ Medicine clinics, followed by Emergency Department and Gynaecology or surgical wards (see Table 2). Other areas included placements in Health Visiting, Haematology, Neurology, Infectious Disease, Treatment rooms, outpatient settings, Macmillan health centre, Orthopaedics, Burns and Dermatology. In terms of student involvement in these areas, open text responses further reported engagement at varying levels, from direct patient discussions (e.g., contraception counselling, HIV care) to observational learning during GUM clinic visits and ward rounds. Other experiences included educational outreach such as promoting safe sex to students, and brief interactions during routine discharge planning or casual ward-based conversations.

The following quotes are representative of the identified ‘Barriers’:

Just talking about it in general ant the stigma around it also because of religion.

*[…] finding the confidence to speak to patients within same age group can be a bit uncomfortable […]*.

Being able to confidentiality explain the importance of sexual health to someone who is possibly older.

I don’t think the issues lies with the lack of knowledge, probably more to do with the lack of know how to engage in a meaningful conversation with patients about their sexual health.

### Finding 2 and 3 knowledge and confidence

**Fig. 2:**
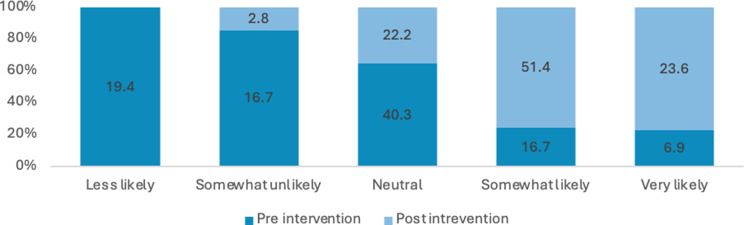
Percentage of responses for how comfortable are you talking to patients about issues related to sexuality

Figure 2 shows the percentages of Likert scale responses for the pre and post intervention. Analysis using Wilcoxon signed rank (paired) test shows statistically significant improvements across all survey responses between pre- and post-intervention with large effect sizes observed for each response. Further, Appendix 2 reports all the z, r and p values in more detail.

### Knowledge

**Fig. 3:**
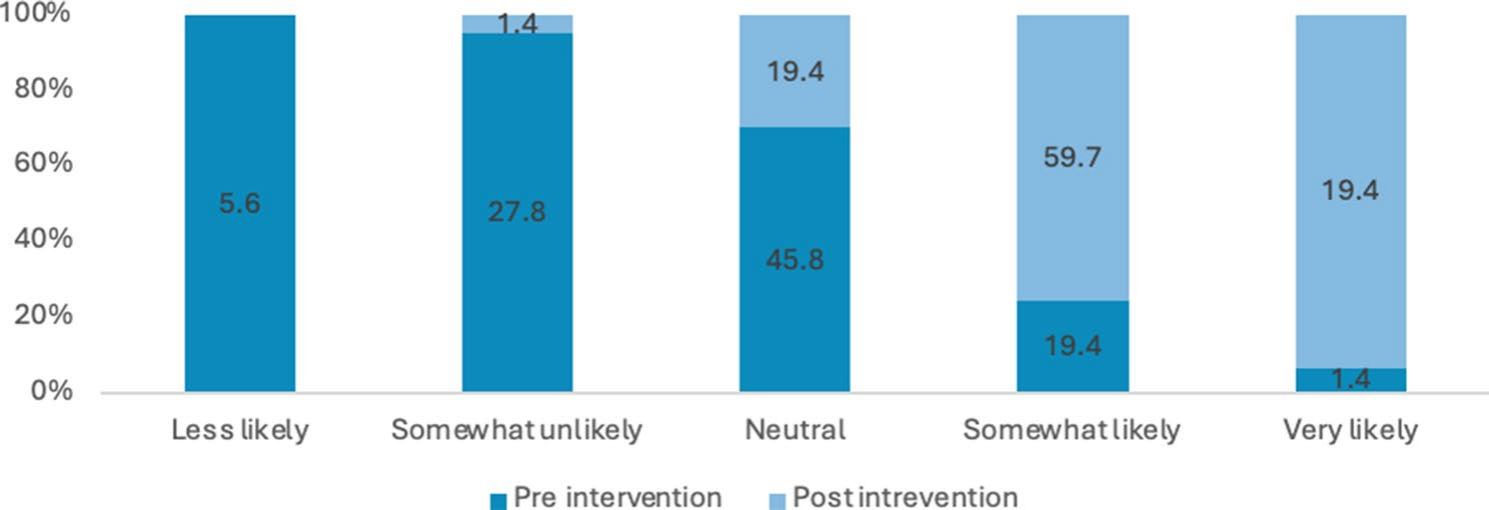
Percentage of responses for how would you rate your knowledge on sexual health?

**Fig. 4:**
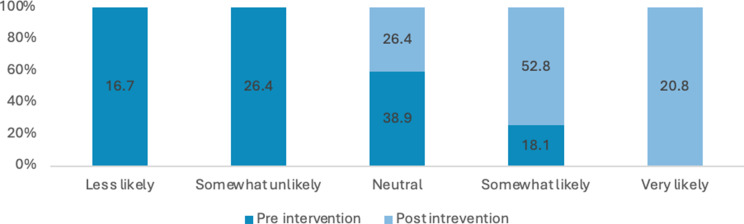
Percentage of responses for how would you rate your knowledge on STIs and their transmission (Chlamydia, gonorrhoea)?

**Fig. 5:**
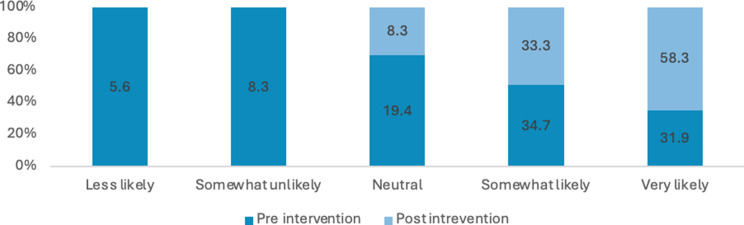
Percentage of responses for how important do you consider asking patients about their sexual health?

**Fig. 6:**
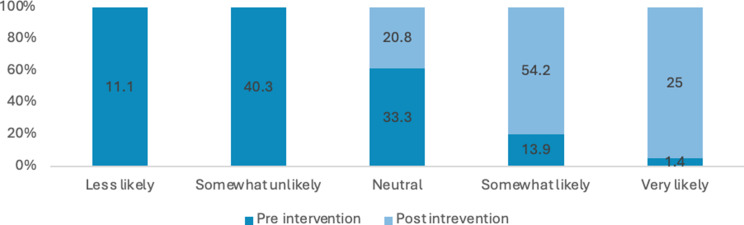
Percentage of responses for how would you rate your knowledge of HIV and its transmission?

Many students reported a lack of comprehensive knowledge prior to attending the educational session, often describing their understanding as general, limited or non-existent. Across these items, there were marked improvements in self-rated knowledge and perceived importance from pre- to post-intervention. The proportion of respondents reporting high knowledge (i.e., “somewhat likely” or “very likely”) of sexual health increased from 21% pre-intervention to 78% post-intervention. Overall, these results indicate substantial gains in knowledge and stronger recognition of the importance of sexual health discussions following the intervention (see Figs. 3, 4, 5 and 6). Specifically, the analysis showed statistically significant improvements were in how students rated the importance of talking to patients about their sexual health (Z=-5.735; *P*<.0010) and ratings of their knowledge in HIV and its transmission (Z=-7.996; *P*<.001) after the lecture and workshop.

The following quotes are representative of students’ perception on ‘Knowledge’:

*Before this session*,* I would suggest all topic*,* sexual and reproductive health not commonly talked about or discussed therefore limited knowledge on it. Very informative session*!

*I think students lack knowledge in STIs and their transmission*,* it’s important to be aware of each of those to provide the best patient care possible*.

*Maybe lack of knowledge around female protection*,* UTIs*,* using pill*.

### Confidence

**Fig. 7:**
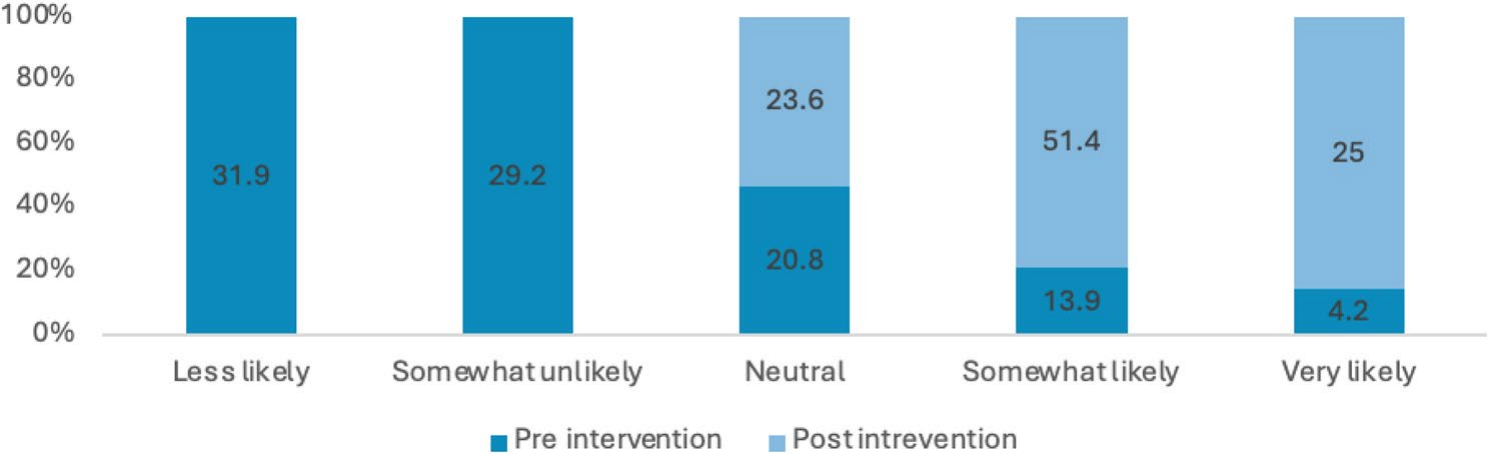
Percentage of responses for How comfortable are you talking to patients about sexually transmitted diseases?

**Fig. 8:**
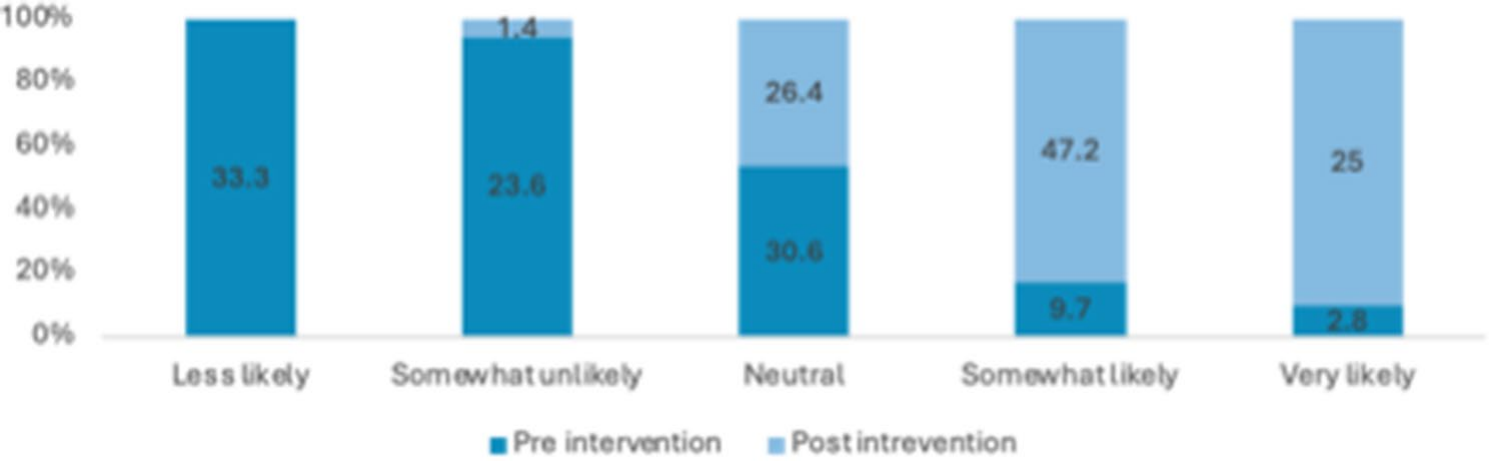
Percentage of responses for how comfortable are you providing education to patients to prevent STIs (Chlamydia, gonorrhoea)?

**Fig. 9:**
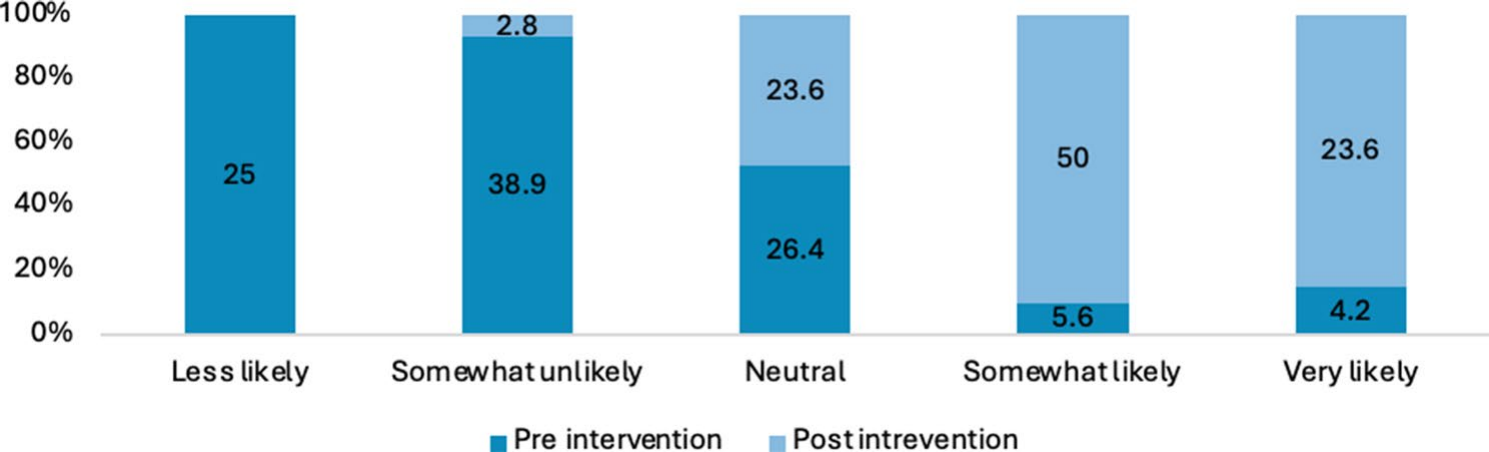
Percentage of responses for how confident are you in providing education to patients to prevent HIV?

**Fig. 10:**
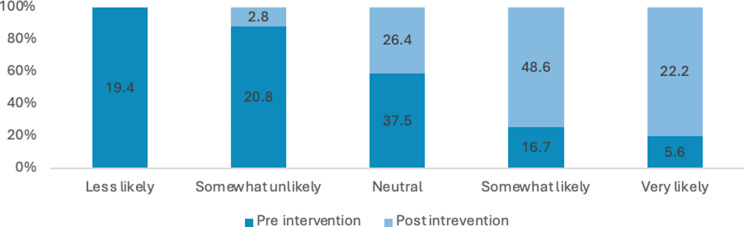
Percenge of responses for how confident are you talking to patients about issues related to sexuality?

**Fig. 11:**
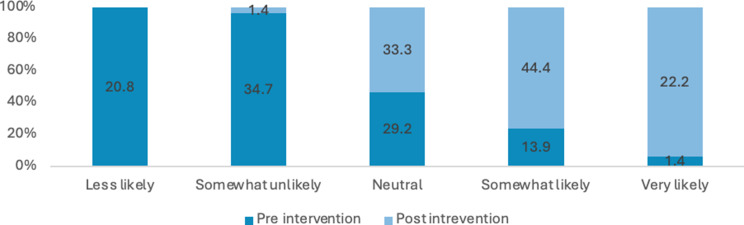
Percentage of responses for how confident are you talking to patients about sexually transmitted diseases?

**Fig. 12:**
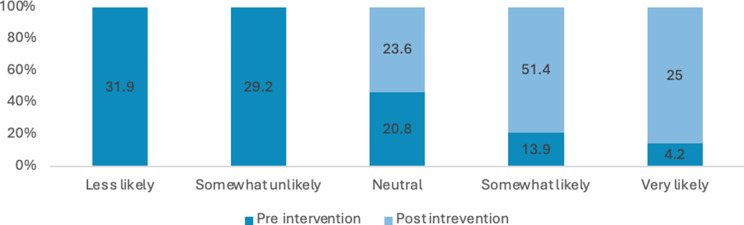
Percentage of responses for how confident are you in providing education to patients to prevent STIs (Chlamydia, gonorrhoea)?

Across all items, there was a substantial increase in the proportion of respondents reporting high comfort or confidence (i.e., “somewhat likely” or “very likely”) from pre-intervention to post-intervention (see Figs. 2, 7, 8, 9, 10, 11 and 12).

Specifically, the comfort levels relating to talking to patients about issues related to sexuality (Z= -7.431; *P*<.001), talking to patients about sexually transmitted diseases (Z=-7.630; *P*<.001), providing education to patients to prevent STIs (Chlamydia, gonorrhoea…) (Z=-8.118; *P*<.001) was statistically significantly improved. Similarly, confidence levels relating to talking to patients about issues related to sexuality (Z=-7.434; *P*<.001), talking to patients about sexually transmitted diseases (Z=-7.706; *P*<.001), providing education to patients to prevent STIs (Chlamydia, gonorrhoea…) (Z=-7.896; *P*<.001) and providing education to patients to prevent HIV (Z=-8.278; *P*<.001) was statistically significantly improved after intervention.

## Discussion

Few interventions have addressed sexual health education within undergraduate nursing programs. Only a small number of curricula have been experimentally evaluated for their effects on nursing students’ knowledge, attitudes, and competencies in meeting patients’ sexual healthcare needs [14]. This study, in line with others, has highlighted that third-year nursing students face different barriers in discussing sexual health issues with patients. These include: a lack of confidence; lack of knowledge about the topic; and lack of exposure [14, 30, 31]. The delivery of a lecture and workshop is associated with improvements overcome some of these barriers and significantly improved their perceptions of comfort, knowledge and confidence in these areas [32].

Our findings highlight, due to insufficient knowledge, student nurses do not feel ready to support patients on these topics [32]. Study findings further report on the range of clinical areas that students are exposed relating to sexual and reproductive health assessment. However, they also reported a lack of opportunities to have conversations with patients, building practice experience and confidence in clinical areas [33]. Nurses have the potential to address different aspects of sexual health with a variety of patients [34, 35]. Therefore, it is important for them to be able to initiate a conversation. Consistent with the findings of this study, existing research suggests that student nurses frequently demonstrate reluctance when discussing sexual health issues with patients [10, 36]. This may contribute to a culture where sexual health topics are not routinely discussed with patients, ultimately not meeting their needs.

To the best of our knowledge, this is the first study to use the 6Ps Framework in to develop an educational intervention tailored to third year adult nursing students in Northern Ireland. Some studies assessed the knowledge and competencies in sexual health, but they did not include a trauma centred approach [37]. The use of The Road Map to Trauma Informed Care [20] along with the 6Ps Framework [6] provided the opportunity to students to explore the different aspects of sexual health; and provided some tips on how to initiate conversations with patients. Our findings indicate that a 2-hour lecture combined with a 2-hour workshop resulted in increased confidence and knowledge among participants. While previous research has reported comparable outcomes, these interventions were typically delivered over a substantially longer duration [11, 13, 14, 30, 38]. This highlights the potential value of brief educational interventions in supporting students’ educational development [12]. However, more studies should be conducted to evaluate the long-term effects of this intervention.

The intervention might have been effective because it drew on evidence that students report higher satisfaction with face-to-face learning, where direct communication enables active engagement [27, 28]. By adopting student-centred approaches and using small tutorial or practical sessions, the intervention supported peer learning and the sharing of knowledge and resources [29]. Although sexual and reproductive health can be a sensitive and potentially controversial topic, addressing these issues through guided, face-to-face discussion helped to manage moral judgements associated with STIs while encouraging constructive debate, which can enhance engagement and reduce discomfort when discussing sexual health [30].

Sexual health remains an important concern for students but their contribution in this area remains largely undiscussed [10]. Future research should focus on assessing the delivery of information to patients around their sexual health [34]. Students have who took part on this study also highlighted the need to focus on developing educational interventions who gives them the opportunity to enhance their trauma-informed skills. These educational interventions should be co-designed with students, practitioners and patients to ensure they address all the relevant areas. University curricula should ensure to include educational resources on sexual and reproductive health.

### Strengths and limitations

This study represents the first known application of the 6Ps framework within a trauma-informed educational approach specifically designed for undergraduate nursing students in the UK. The use of a mixed-methods design provided a comprehensive evaluation, combining quantitative measurement of changes in student confidence and knowledge with qualitative exploration of perceived barriers and learning experiences.

However, the study was conducted at a single institution and involved only adult nursing students, which may limit the generalisability of the findings. Other fields of nursing were also not represented; children and young people nursing, mental health and learning disabilities. Students bring diversity through their personal experiences and backgrounds which may have impacted how the intervention was interpreted. Participation was encouraged during class time; however, self-selection bias may still have occurred. Students who chose to complete both questionnaires may have had a greater interest in sexual health etc. Although the study sought to mitigate discomfort through a digital survey and emphasis on voluntary participation, some students may have opted out due to embarrassment or discomfort.

This may limit the representativeness of the sample and reduce the generalisability of the findings to the wider student population. The absence of a control group restricts the ability to determine whether the observed changes were a direct result of the intervention or influenced by other factors, such as ongoing clinical experience, parallel teaching within the programme, or students’ independent learning.

The study relied exclusively on self-reported data. Self-report measures are vulnerable to several forms of bias, including inaccuracies in recall, overestimation or underestimation of learning, and social desirability bias, particularly given the sensitive nature of sexual health.

Finally, the study did not include long-term follow-up. As a result, it is unclear whether the improvements identified were sustained over time or translated into meaningful changes in clinical practice.

## Supplementary Information

Below is the link to the electronic supplementary material.

Supplementary Material 1

## Data Availability

Data are available upon reasonable request. If there is a reasonable request, deidentified participant data used in the research are available via emailing the corresponding author after publication.

## References

[1] Who. Sexual health. 2006. https://www.who.int/health-topics/sexual-health#tab=tab_2. Accessed 26 Jun 2025.

[2] Verrastro V, Saladino V, Petruccelli F, Eleuteri S. Medical and health care professionals’ sexuality education: state of the art and recommendations. Int J Environ Res Public Health. 2020;17. 10.3390/IJERPH17072186. 10.3390/ijerph17072186 PMC7177861 32218258

[3] Roper Nancy L, Winifred TA. The roper-logan-tierney model of nursing: based on activities of living / Nancy Roper, Winifred W. Logan, Alison J. Tierney ; foreword by Ann Marriner Tomey. Churchill Livingstone; 2000.

[4] Sung SC, Lin YC. Effectiveness of the sexual healthcare education in nursing students’ knowledge, attitude, and self-efficacy on sexual healthcare. Nurse Educ Today. 2013;33:498–503. 10.1016/j.nedt.2012.06.019. 22789872 10.1016/j.nedt.2012.06.019

[5] Davis Sally. Rehabilitation: the use of theories and models in practice. 1st edition. Edinburgh: Elsevier Churchill Livingstone; 2005.

[6] National Coalition for Sexual Health. Sexual health questions to ask all patients. 2021. https://www.nationalcoalitionforsexualhealth.org/tools/for-healthcare-providers/sexual-health-questions-to-ask-all-patients/. Accessed 26 Jun 2025.

[7] Shindel AW, Parish SJ. Sexuality Education in North American Medical Schools: Current Status and Future Directions (CME). J Sex Med. 2013;10:3–18. 10.1111/J.1743-6109.2012.02987.X. 23343168 10.1111/j.1743-6109.2012.02987.x

[8] Prize NBT, Shimony-Kanat S, Wruble ACKW. Gaps in sexual health content of healthcare professional curriculum: a systematic review of educational interventions. BMC Med Educ. 2023;23:1–25. 10.1186/S12909-023-04901-1/FIGURES/1. 38062394 10.1186/s12909-023-04901-1 PMC10704846

[9] Uzdavines A, Helmer DA, Spelman JF, Mattocks KM, Johnson AM, Chardos JF, et al. Sexual Health Assessment Is Vital to Whole Health Models of Care. JMIRx med. 2022;3:e36266. 10.2196/36266. 37725523 10.2196/36266 PMC10414374

[10] Blakey EP, Aveyard H. Student nurses’ competence in sexual health care: a literature review. J Clin Nurs. 2017;26:3906–16. 10.1111/JOCN.13810. 28328169 10.1111/jocn.13810

[11] Ross MW, Leshabari S, Rosser BRS, Trent M, Mgopa L, Wadley J, et al. Evaluation of an assessment instrument for a sexual health curriculum for nurses and midwifery students in Tanzania: The sexual health education professionals scale (SHEPS). Appl Nurs Res. 2018;40:152–6. 10.1016/j.apnr.2018.01.005. 29579491 10.1016/j.apnr.2018.01.005 PMC8127620

[12] White BP, Abuelezam NA, Dwyer AA, Fontenot HB. A sexual health course for advanced practice registered nurses: effect on preparedness, comfort, and confidence in delivering comprehensive care. Nurse Educ Today. 2020;92. 10.1016/j.nedt.2020.104506. 10.1016/j.nedt.2020.104506 32599471

[13] Gündüz CS, Demirci N. The Effects of Distance Sexual Health Education Based on the PLISSIT Model on Knowledge, Attitude, And Self-Efficacy in Nursing Students: A Randomized Controlled Trial. Int J Sex Health. 2025. 10.1080/19317611.2025.2573689.

[14] Coleman DC, Frederick A, Cron S, Markham C, Guilamo-Ramos V, Santa Maria D. Impact of preparing nursing students to deliver a parent-based sexual health intervention on attitudes and intentions for sexual health education and parent communication counseling: a mixed methods study. BMC Nurs. 2023;22. 10.1186/s12912-023-01531-2. 10.1186/s12912-023-01531-2 PMC10563268 37817237

[15] NMC. (Nursing and Midwifery Council). The Code. 2018.

[16] Forero R, Nahidi S, De Costa J, Mohsin M, Fitzgerald G, Gibson N, et al. Application of four-dimension criteria to assess rigour of qualitative research in emergency medicine. BMC Health Serv Res. 2018;18:1–11. 10.1186/s12913-018-2915-2. 29454350 10.1186/s12913-018-2915-2 PMC5816375

[17] Lincoln YS, Guba EG. Naturalistic Inquiry. Sage Publication; 1985.

[18] Hadi MA, José Closs S. Ensuring rigour and trustworthiness of qualitative research in clinical pharmacy. Int J Clin Pharm. 2016;38:641–6. 10.1007/s11096-015-0237-6. 26666909 10.1007/s11096-015-0237-6

[19] Nowell LS, Norris JM, White DE, Moules NJ. Thematic Analysis: Striving to Meet the Trustworthiness Criteria. Int J Qualitative Methods. 2017. 10.1177/1609406917733847.

[20] Clinical Training Center for Sexual and reproductive Health. Clinician guide for trauma-informed care. Kansas City; 2022.

[21] WHO. Sexual health. 2006. https://www.who.int/health-topics/sexual-health#tab=tab_2. Accessed 18 Feb 2026.

[22] Townsend HF, Hobbs JR, Cole H. Assessing nursing students’ confidence in sexual health counseling and knowledge. Teach Learn Nurs. 2024;19:e101–5. 10.1016/J.TELN.2023.09.012.

[23] Ryan KL, Arbuckle-Bernstein V, Smith G, Phillips J. Let’s talk about sex: a survey of patients’ preferences when addressing sexual health concerns in a family medicine residency program office. PRiMER. 2018;2. 10.22454/PRIMER.2018.728252 10.22454/PRiMER.2018.728252 PMC7426112 32818195

[24] Hsieh H-F, Shannon SE. Three approaches to qualitative content analysis. 2005. 10.1177/1049732305276687 10.1177/1049732305276687 16204405

[25] Bengtsson M. How to plan and perform a qualitative study using content analysis. NursingPlus Open. 2016;2:8–14. 10.1016/j.npls.2016.01.001.

[26] O’Cathain A, Murphy E, Nicholl J. The quality of mixed methods studies in health services research. J Health Serv Res Policy. 2008;13:92–8. 18416914 10.1258/jhsrp.2007.007074

[27] Creswell JW, Klassen AC, Plano Clark VL, Clegg Smith K. Best practices for mixed methods research in the health sciences. 2011.

[28] Ziegler N, Kang L. Mixed methods desings. In: Moeller AJ, Creswell JW, Saville N, editors. Second Language Assessment and Mixed Methods Research. Camridge: Cambridge University Press; 2016.

[29] Fosnacht K, Sarraf S, Howe E, Peck LK. How important are high response rates for college surveys? Rev High Ed. 2017;40:245–65. 10.1353/RHE.2017.0003.

[30] Ünal Toprak F, Turan Z. The effect of sexual health courses on the level of nursing students’ sexual/reproductive health knowledge and sexual myths beliefs in Turkey: A pretest-posttest control group design. Perspect Psychiatr Care. 2021;57:667–74. 10.1111/ppc.12593. 32730656 10.1111/ppc.12593

[31] Seid K, Kebede T, Dessalegn N, Ejara Y, Moga F, Daniel M, et al. Nursing students’ attitudes and beliefs regarding sexual healthcare in Ethiopia: an online cross-sectional study. PLoS ONE. 2022;17. 10.1371/journal.pone.0278760. 12 December. 10.1371/journal.pone.0278760 PMC9728890 36477268

[32] Jadoon SB, Nasir S, Victor G, Pienaar AJ. Knowledge attitudes and readiness of nursing students in assessing peoples’ sexual health problems. Nurse Educ Today. 2022;113. 10.1016/j.nedt.2022.105371. 10.1016/j.nedt.2022.105371 35524991

[33] Saus-Ortega C, Ballestar-Tarín ML, Chover-Sierra E, Martínez-Sabater A. Contents of the sexual and reproductive health subject in the undergraduate nursing curricula of spanish universities: a cross-sectional study. Int J Environ Res Public Health. 2021;18. 10.3390/IJERPH182111472/S1. 10.3390/ijerph182111472 PMC8583184 34769987

[34] Annerstedt CF, Glasdam S. Nurses’ attitudes towards support for and communication about sexual health—A qualitative study from the perspectives of oncological nurses. J Clin Nurs. 2019;28:3556–66. 10.1111/jocn.14949. 31165516 10.1111/jocn.14949

[35] Rubio-Rask SE, Farver-Vestergaard I, Hilberg O, Løkke A. Sexual health communication in COPD: the role, contents and design of patient information leaflets. Chron Respir Dis. 2021;18. 10.1177/14799731211020322. 10.1177/14799731211020322 PMC8252346 34189938

[36] Ślusarska B, Marcinowicz L. Nursing and midwifery students’ attitudes towards addressing patient sexual health in their future profession: polish adaptation and validation of the students’ attitudes towards addressing sexual health extended questionnaire (SA-SH-Ext). PLoS ONE. 2024;19. 10.1371/journal.pone.0300515. 6 June. 10.1371/journal.pone.0300515 PMC11192396 38905205

[37] Martínez-Galiano JM, Gonzalez-Cabrera M, Rodriguez-Almagro J, Hernández-Martínez A. Evaluation of knowledge and competencies in sexual and reproductive health care using an escape room with scenario simulations. Nursing Reports. 2024, Vol 14, Pages 683–694. 2024;14:683–94. 10.3390/NURSREP14020052 10.3390/nursrep14020052 PMC10961789 38525698

[38] Sarpkaya Güder D, Tekbaş S. The effect of sexual health course on students’ level of belief in sexual myths. Int J Sex Health. 2022;34:267–76. 10.1080/19317611.2021.2005730. 38596524 10.1080/19317611.2021.2005730 PMC10903675

