# Supplementary material for: Evaluating nursing student confidence after sexual health training workshop
Source: BMC Nurs. 2026 Mar 5;25:351. doi: 10.1186/s12912-026-04509-y (PMC13072520; doi:10.1186/s12912-026-04509-y)
Supplement: Supplementary file 1 — Supplementary Material 1 [file 12912_2026_4509_MOESM1_ESM.docx]

Appendix 1- Survey questions

| Please indicate your age range | 18-24  25-30  31-36  37-40  41-50  50+ |  |
| --- | --- | --- |
| Please indicate your gender | Male  Female  Transgender  Non-binary  Genderqueer  Gender fluid  Other (please indicate) |  |
| Have you received training on sexual health to date? | Yes/No | Developed by research team |
| If yes, please provide any further details e.g., content, resources, location if possible | Text box | Develop by research team |
| To date, have you had the opportunity to discuss sexual health with patients while on clinical placement? | Yes/No | Develop by research team |
| If yes, please indicate where | Text box | Developed by research team |
| How **comfortable** are you talking to patients about issues related to sexuality? | Likert Scale (1-5) | Adapted from Mahabamunuge et al., 2021 |
| How **comfortable** are you talking to patients about sexually transmitted diseases? | Likert Scale (1-5) | Adapted from Mahabamunuge et al., 2021 |
|  |  |  |
| How **comfortable** are you **providing education** to patients to prevent STIs (Chlamydia, gonorrhoea…)? | Likert Scale (1-5) | Developed by research team |
| How **confident** are you talking to patients about issue related to sexuality? | Likert Scale (1-5) | Developed by research team |
| How **confident** are you talking to patients about sexually transmitted diseases? | Likert Scale (1-5) | Developed by research team |
| How **confident** are you in providing education to patients to prevent STIs (Chlamydia, gonorrhoea…)? | Likert Scale (1-5) | Developed by research team |
| How **confident** are you in providing education to patients to prevent HIV? | Likert Scale (1-5) | Developed by research team |
| How would you **rate your knowledge** on sexual health? | Likert Scale (1-5) | Developed by research team |
| How would you **rate your knowledge** on STIs and their transmission (Chlamydia, Gonorrhoea ..) | Likert Scale (1-5) | Developed by research team |
| How would you **rate your knowledge** on HIV and its transmission? | Likert Scale (1-5) | Developed by research team |
| How important do you consider asking patients about their sexual health? | Likert Scale (1-5) | Developed by research team |
| What are the barriers that may prevent you to initiate a conversation on sexual health with patients (tick as many as you like) | - Confidence - Time - Embarrassment - Concern about keeping information private - Knowledge - My practice supervisor does not support me in this - Privacy - Culture - Religious belief - No barriers, I would initiate a conversation - Other (text box) | Adapted from Ryan e al. 2018 |
| What impact did the lecture + workshop have on your confidence in discussing sexual health with patients? | Likert Scale (1-5) | Developed by research team |
| What impact did the lecture + workshop have on your knowledge of sexual health? | Likert Scale (1-5) | Developed by research team |
| What, if any, topics of sexual and reproductive health do students lack knowledge? | Text box | (Adapted from) Townsend et al. 2024 |
| What, if any, topics of sexual and reproductive health do students lack confidence? | Text box | (Adapted from) Townsend et al. 2024 |

Appendix 2- Statistical analysis

|  | Z, p, r |
| --- | --- |
| How comfortable are you talking to patients about issues related to sexuality? | Z= -7.431,P<.001; -.75 |
| How comfortable are you talking to patients about STDs? | Z=-7.630, P<.001, r=-.77 |
| How comfortable are you providing education to patients to prevent STIs (e.g. Chlamydia etc). | Z=-8.118, P<.001, r=-.82 |
| How confidence are you in providing education to patients to prevent HIV? | Z=-7.434, P<.001, r=-.75 |
| How confidence are you talking to patients about issues related to sexuality? | Z=-7.706, P<.001, -.78 |
| How confident are you talking to patients about STDs? | Z=-7.896, P<.001, -.80 |
| How confident are you in providing education to patients to prevent STIs (e.g. Chlamydia etc) ? | Z=-8.278, P<.001, r=-.84 |
| How would you rate your knowledge on sexual health? | Z=-7.606, P<.001, r=-.77 |
| How would you rate your knowledge on STIs and their transmission (e.g. Chlamydia etc)? | Z=-7.684, P<.001, r=-.78 |
| How important do you consider asking patients about their sexual health? | -5.735, P<.001, r=-.58 |
| How would you rate your knowledge on HIV and its transmission? | Z=-7.996, P<.001, r=-.81 |
